# Centromere and Pericentromere Transcription: Roles and Regulation … in Sickness and in Health

**DOI:** 10.3389/fgene.2018.00674

**Published:** 2018-12-21

**Authors:** Ksenia Smurova, Peter De Wulf

**Affiliations:** Centre for Integrative Biology, University of Trento, Trento, Italy

**Keywords:** centromere, pericentromere, kinetochore, heterochromatin, long non-coding RNA, transcription

## Abstract

The chromosomal loci known as centromeres (CEN) mediate the equal distribution of the duplicated genome between both daughter cells. Specifically, centromeres recruit a protein complex named the kinetochore, that bi-orients the replicated chromosome pairs to the mitotic or meiotic spindle structure. The paired chromosomes are then separated, and the individual chromosomes segregate in opposite direction along the regressing spindle into each daughter cell. Erroneous kinetochore assembly or activity produces aneuploid cells that contain an abnormal number of chromosomes. Aneuploidy may incite cell death, developmental defects (including genetic syndromes), and cancer (>90% of all cancer cells are aneuploid). While kinetochores and their activities have been preserved through evolution, the CEN DNA sequences have not. Hence, to be recognized as sites for kinetochore assembly, CEN display conserved structural themes. In addition, CEN nucleosomes enclose a CEN-exclusive variant of histone H3, named CENP-A, and carry distinct epigenetic labels on CENP-A and the other CEN histone proteins. Through the cell cycle, CEN are transcribed into non-coding RNAs. After subsequent processing, they become key components of the CEN chromatin by marking the CEN locus and by stably anchoring the CEN-binding kinetochore proteins. CEN transcription is tightly regulated, of low intensity, and essential for differentiation and development. Under- or overexpression of CEN transcripts, as documented for myriad cancers, provoke chromosome missegregation and aneuploidy. CEN are genetically stable and fully competent only when they are insulated from the surrounding, pericentromeric chromatin, which must be silenced. We will review CEN transcription and its contribution to faithful kinetochore function. We will further discuss how pericentromeric chromatin is silenced by RNA processing and transcriptionally repressive chromatin marks. We will report on the transcriptional misregulation of (peri)centromeres during stress, natural aging, and disease and reflect on whether their transcripts can serve as future diagnostic tools and anti-cancer targets in the clinic.

## Centromeres, Kinetochores, and Aneuploidy

**FIGURE 1:**
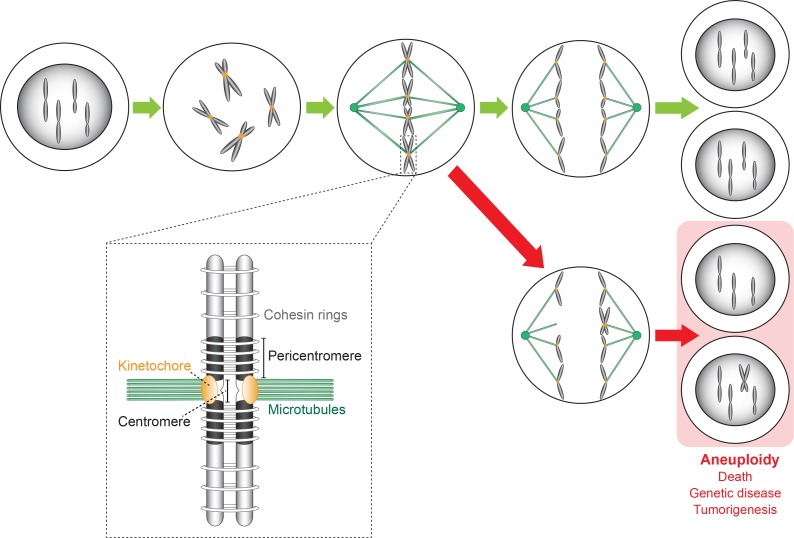
Chromosome replication and segregation in a cell undergoing the mitotic cell division cycle. Kinetochores bi-orient the replicated chromosomes (forming sister chromatids) on the metaphase spindle along which they then segregate in opposite directions into the two daughter cells that receive a full complement of the maternal genome (green arrows). Errors made during the segregation process caused by CEN or kinetochore malfunction lead to aneuploid daughter cells (red arrows) carrying an abnormal number of chromosomes. Consequences are cell death, genetic disease (developmental defects), and cancer initiation/progression. The insert shows a more detailed representation of a sister chromosome pair whose chromosomes (original and copy) are linked by cohesion rings. The sister chromosomes are bound to the spindle microtubules via kinetochores that assemble on the CEN sequence of each chromosome.

During cell division, the replicated chromatids that are associated by cohesin rings bind to the microtubules of the metaphase spindle, which extend from two opposite spindle poles (Figure 1). This binding is mediated by kinetochores, each of which assembles on the centromere (CEN) of each chromatid. CENP-A/CenH3, a variant of histone protein H3, recruits all kinetochore subunits and spindle assembly checkpoint (SAC) proteins to the centromeric nucleosome(s). To prevent aneuploidy, the SAC monitors chromosome-spindle attachment at each kinetochore. The SAC arrests the cell division process at the metaphase–anaphase transition when a single chromosome pair is found to be unbound or misbound to the mitotic spindle. The SAC kinase Aurora B then phosphorylates the outer kinetochore Ndc80 protein of each misbound sister pair to detach it from the spindle structure. The delay of mitosis allows for a correct re-attachment. Only when the SAC is satisfied will all sister chromosomes separate by enzymatic cleavage of the cohesin rings. Each kinetochore-bound chromatid then moves into the daughter cells by depolymerization of the spindle microtubules and, in some eukaryotes, by additonal motor protein activity. In the end, each cell receives a full complement of the maternal genome (Figure 1). Abnormal CEN or kinetochore activity has been linked with cancer initiation/progression, developmental defects, and genetic disease (Holland and Cleveland, 2009; Santaguida and Amon, 2015). For more detailed information about kinetochores we refer to Fukagawa and Earnshaw (2014); McKinley and Cheeseman (2016); and Musacchio and Desai (2017). Of note, during revision of this manuscript, an excellent review was published (Perea-Resa and Blower, 2018) partially overlaps with ours in subject matter.

## Centromeres: Evolutionary Diverged Sequences

**FIGURE 2:**
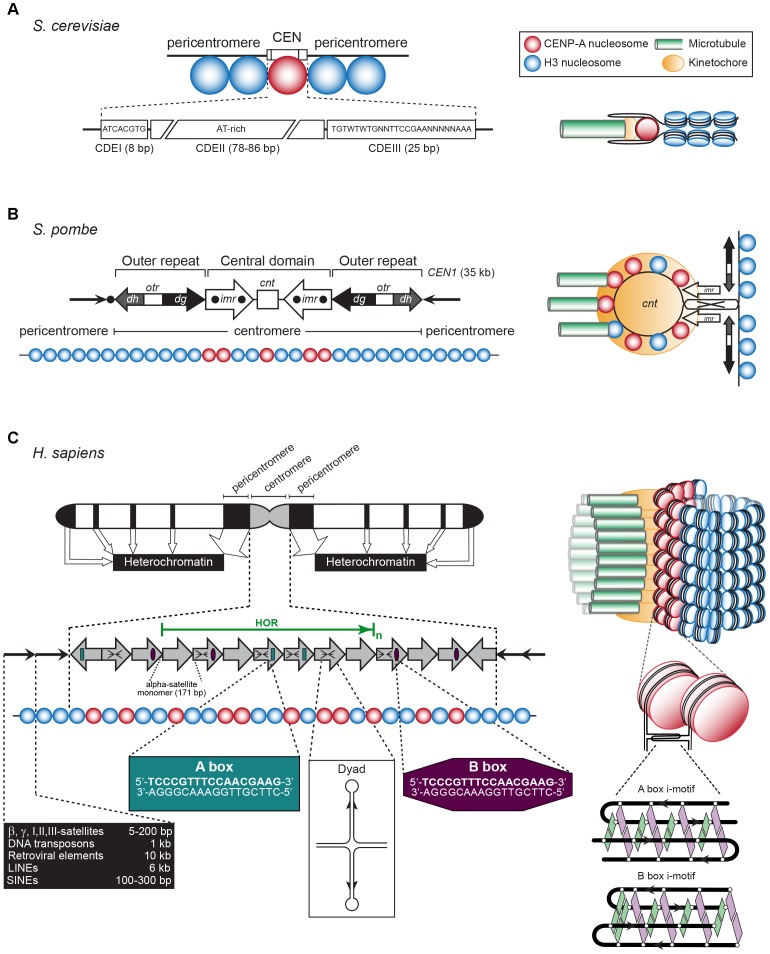
**(A)** Left: The *S. cerevisiae* point CEN (the consensus CDEI and CDIII sequences are indicated; W = A or T, N = any base). Right: A single CENP-A containing nucleosome is bound to a single microtubule by a single kinetochore (based on Bloom and Costanzo, 2017). **(B)** Left: The *S. pombe* regional CEN. Black dots: tRNA clusters. See text for details. Right: A single, looped CEN harboring CENP-A- and histone H3-containing nucleosomes is bound to three microtubules via a single kinetochore (based on McFarlane et al., 2010). **(C)** Left: A typical human (*Homo sapiens*) chromosome. White regions: euchromatin, gray region: centromeric chromatin, black regions: heterochromatin. The latter represent the pericentromeres, telomeres, LINEs, SINEs, micro- and macrosatellites, β, γ, I, II, III-satellites, rDNA, and DNA transposons (approximate lengths are indicated in the black box). The gray arrows represent the CEN alpha-satellite monomers, organized in a head-to-tail fashion. HOR, high-order repeat of alpha-satellite monomers (green arrow). A-boxes (dark green) and B-boxes (purple) are indicated, as well as the cruciform configuration of a dyad sequence. Right: Human centromeric chromatin with the CENP-A containing nucleosomes clustered and exposed in amphipathic configuration at its outside is bound by numerous kinetochores to a bundle of microtubule fibers (based on Fukagawa and Earnshaw, 2014). See text for details.

The CEN was first identified as the central constriction of each chromosome during the light microscopic analysis of mitotic salamander cells (Flemming, 1880). Today, it is defined as the chromosomal region that underlies the stable transmission of the nuclear genomic content from one generation to the next. In the 1980s; the CEN of budding yeast *Saccharomyces cerevisiae* chromosome 3, and all three CEN of the fission yeast *Schizosaccharomyces pombe* were the first CEN loci to be characterized (Clarke and Carbon, 1980; Nakaseko et al., 1987; Figures 2A,B). The short budding yeast “point” CEN is ∼120 bp long and contains three DNA elements that wrap around a single CEN nucleosome. Alternate stretches of A and T residues, which cause DNA bending, comprise CDEII, which is bordered by palindromic motifs named CDEI and CDEIII (Figure 2A). In contrast to CDEII and CDEIII, CDEI is not essential for kinetochore activity but mutations in its sequence cause chromosome loss (Niedenthal et al., 1991). In *S. cerevisiae*, the CEN sequence *per se* defines CEN identity. In contrast and because of their 40–110 kb length, the CEN in fission yeast are designated as “regional.” They comprise a 4–7 kb core sequence named *cnt* that encloses multiple CEN nucleosomes. The core is flanked by inverted, 6 kb-long innermost *imr* repeats that contain clusters of tRNA genes. Together, these three elements form the central domain, which is flanked left and right by outer repeats, *otr*, named *dg* and *dh* (Figure 2B).

The regional CEN of most higher eukaryotes are comprised of retrotransposon repeats and repeats of a simple 171-bp CEN sequence, named alpha-satellite DNA, where the CENP-A nucleosomes reside (Figure 2C). The surrounding pericentromeric domains contain repeats that are less ordered. In humans and most primates, the alpha-satellite sequence is organized in back-to-back fashion, forming a high-order repeat (HOR) (Manuelidis and Wu, 1978; Willard, 1985). Within a HOR, alpha-satellite monomers are 50–70% identical (Willard, 1985). Each HOR is repeated hundreds-to-thousands of times, producing 2–5 Mb-long arrays (Aldrup-MacDonald and Sullivan, 2014; Figure 2C). Different chromosomes are distinguished by variations within the alpha-satellite sequences, by the number of alpha-satellite monomers, and the overall size of the HOR. Not all alpha-satellite monomers contribute to human kinetochore activity, these are labeled as “inactive.” Human CEN contain alpha-satellite monomers of the A and B type, while lower primates only have A-type satellites (Alexandrov et al., 2001). Both monomers differ in a 17-bp sequence called A or B box (Figure 2C). The latter, also named CENP-B box, binds CEN protein CENP-B (Masumoto et al., 1989). It is unclear if a specific protein binds to the A box. Human chromosomes, except the Y chromosome, contain B-type alpha-satellite monomers (Tyler-Smith and Brown, 1987). A third type of alpha-satellite monomers contains neither an A nor a B box. The CEN in mice consist of homogeneous arrays of 120-bp minor satellite (MinSat) repeats, that are flanked by repeats of less-ordered 234-bp major gamma-satellite (MajSat) sequences (Joseph et al., 1989). The CEN repeat units in higher eukaryotes are typically around 150 bp in length [178 bp in plants (Kumekawa et al., 2001; Nagaki et al., 2003)], each enclosing one CENP-A nucleosome. However, they can be much shorter as in *Drosophila melanogaster*, whose CEN (200–500 kb) are made up of 10-bp repeats followed by 11/12-bp tandem repeats (Garavís et al., 2015b).

Most eukaryotes are monocentric since their chromosomes contain one CEN. In contrast, moths and butterflies, as well as nematodes such as *Caenorhabditis elegans*, and arachnids contain holocentric CEN that cover the entire chromosome, except for the telomeric regions (Heckmann et al., 2011; Steiner and Henikoff, 2014). While the *C. elegans* genome comprises few tandem repeats (Hillier et al., 2007), ∼50% of the genome is associated with CENP-A in 20 CEN domains of variable size (Albertson and Thomson, 1982; Gassmann et al., 2012). Its kinetochores hence may assemble randomly or at specific regions. While the evolutionary forces that drove holocentrism are unknown, one benefit may lie in DNA breaks. In contrast to broken monocentric chromosomes, fragmented holocentric chromosomes can still segregate in mitotic anaphase because of the multiple microtubule attachments they may contain. Nevertheless, the prevalence of monocentrism suggests selective advantages, possibly related to difficulties in segregating recombined holocentric chromosomes during meiosis (Maddox et al., 2004). For more detailed information about CEN we refer to Aldrup-MacDonald and Sullivan (2014); Bloom and Costanzo (2017); and Fukagawa and Earnshaw (2014).

## Transcriptionally Enhanced Centromere Features

Centromeres evolved rapidly due to homologous recombinations between stretches of tandemly repeated sequences. Even within one organism CEN sequences differ significantly between its chromosomes. Despite this divergence, most CEN-binding kinetochore proteins are conserved. This “CEN paradox” is explained by the maintenance of CEN-specific structural themes during the co-evolution of CEN DNA and the CEN-binding kinetochore proteins (Henikoff et al., 2001). The adaptive evolution of CENP-A and its orthologs involves regions within this protein that are predicted to contact the centromeric DNA (Talbert et al., 2004; Schueler et al., 2010). In turn, CEN may not have been selected based on their DNA sequence but rather on non-canonical structures that act as beacons for kinetochores and sustain the pulling forces that CEN nucleosomes undergo during chromosome segregation. Studies of CEN from numerous species have indicated a functional significance of non-B-form DNA structures including single-stranded (ss) DNA, hairpins, triplexes, i-motifs, and cruciform extrusions as observed *in vitro* and/or *in vivo* (Zhu et al., 1996; Ohno et al., 2002; Jonstrup et al., 2008; Garavís et al., 2015a,b; Aze et al., 2016; Kabeche et al., 2018). All CEN, except those of *S. cerevisiae*, maintain a high level of inter-repeat sequence property, suggestive of a recombination-based mechanism that produces covalently closed stem–loop structures, which may define CEN recognition and activity. A conserved stem–loop model would demand repeat DNA sequences, explaining the evolution of the CEN’s repeat-array configuration (illustrated for the *S. pombe* CEN in Figure 2B). Metazoans might require a threshold number of these loop structures to produce a functional CEN (McFarlane et al., 2010). Possibly, the single-stranded loops could be formed temporarily during replication and/or transcription to seed kinetochores.

A neocentromere, being a new CEN that originates at a site that is not centromeric usually due to disruption of the natural CEN, lack centromeric alpha-satellite DNA, but are fully competent to generate a primary constriction and assemble a functional kinetochore (Marshall et al., 2008) indicating that alpha-satellite DNA *per se* is not a trigger for attracting CEN proteins. However, neocentromeres actually form at chromosomal sites that not only contain pre-existing repeats but further develop extensive repetitive DNA sequences over time, indicating the advantage of acquiring an extensive repeat configuration (Marshall et al., 2008). Epigenetic mechanisms are additionally required for maintaining neocentromere identity and activity.

*Drosophila melanogaster* CEN are made up of short satellite DNA repeats (AATAACATAG)*_n_* followed by doceda tandem repeats (CCCGTACT[C]GGT) that show an asymmetric distribution of G and C residues. *In vitro*, the C-rich dodeca satellite single strand produces an “i-motif”; a cubic structure that is formed by the head-to-tail association of two parallel strands combined in antiparallel fashion (Garavís et al., 2015b; Figure 2C). Similar i-motif structures arise *in vitro* between human alpha-satellite monomers in which the C-rich strand of one A-box associates with that of a neighboring A-box. CEN-B boxes also form i-motifs, while those produced from an A- and B-box strand are somewhat unstable *in vitro* (Garavís et al., 2015b). Murine Y CEN satellite DNA that lacks an A/B-box has a sequence capable of forming an i-motif in an equivalent position (Garavís et al., 2015a). As i-motifs can form upon transcriptionally induced supercoiling (Sun and Hurley, 2009) and since the transcription of alpha-satellite DNA is required for CEN function (Chan et al., 2012), negative superhelicity may favor i-motif formation under physiological conditions.

*In vivo* evidence for the phasing of CENP-A nucleosomes showed that their positioning is a physical requirement for CEN function (Hasson et al., 2013; Zhang et al., 2013). In most higher eukaryotes CEN chromatin contains blocks of CENP-A that are interspersed with blocks of histone H3-containing nucleosomes (Bodor et al., 2014; Fukagawa and Earnshaw, 2014; Figures 2A,C). CENP-A nucleosomes may associate laterally and exclude the H3-containing nucleosomes. The flexibility observed in the chromatin that flanks the CENP-A nucleosomes facilitates these interactions (Panchenko et al., 2011; Hasson et al., 2013). In humans, the phasing of CENP-A nucleosomes on alpha-satellite DNA places the A- and B-boxes at the beginning and at the end of the nucleosome (Hasson et al., 2013). Models of CEN chromatin folding into an amphipathic helix, loop, or boustrophedon that expose the CENP-A nucleosomes at the chromatin surface have been suggested to facilitate kinetochore formation (Blower et al., 2002; Bloom and Costanzo, 2017). A hierarchical mechanism of chromatin folding based on A- and B-box interactions and i-motif formation may determine the 3D organization of the CEN. Although CENP-B null mice are viable (Kapoor et al., 1998), CENP-B is required for *de novo* CEN formation on artificial chromosomes (Ohzeki et al., 2002) and enhances chromosome segregation fidelity (Fachinetti et al., 2015). Possibly, B-box i-motifs contribute to a nucleosome environment that improves kinetochore assembly and activity.

While examining the CEN from different species, Kasinathan and Henikoff (2018) identified clade-specific variations in <10-bp dyad symmetries predicted to adopt stable non-B-form cruciform extrusions (Figure 2C). Satellites lacking CENP-B boxes were highly enriched in these palindromes. Non-B-form DNA regions were abundant in human alpha-satellite and murine MinSat sequences from activated B cells, while reduced levels were observed in non-proliferating cells, suggesting that replication induces cruciform extrusions at CEN in dividing cells (Kasinathan and Henikoff, 2018). The authors propose that CEN are either highly enriched with dyad sequences or less-enriched in dyads that flank a nearby binding site for a DNA-bending protein whose association may stimulate dyad cruciform formation. The four-way junctions of the cruciform could be recognized by the HJURP chaperone (Scm3 in yeast) that loads CENP-A into the centromeric nucleosome (Dunleavy et al., 2009; Foltz et al., 2009; Sanchez-Pulido et al., 2009). Non-B form elements may also facilitate CEN transcription initiation and elongation by RNA polymerase II (RNAPII), enabling the loading of CENP-A during nucleosome remodeling. Also, CENP-B may be dispensable for CEN where HJURP is recruited by CENP-C and the MIS18 complex (Nardi et al., 2016) (see below). Hence, A/B boxes and dyad sequences may organize and activate CENP-A loading into CEN nucleosomes.

## Post-Translational Modifications of Centromeric and Pericentromeric Chromatin

**FIGURE 3:**
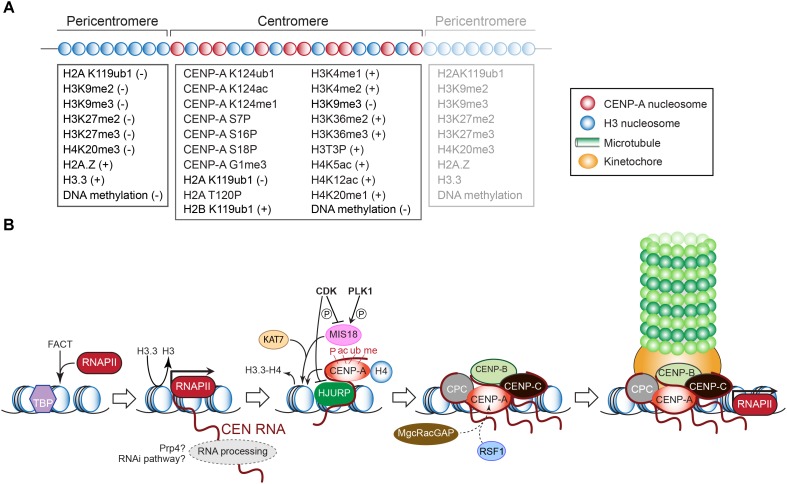
**(A)** Epigenetic modifications that mark histones and DNA (cytosines) in the pericentric and centromeric domains. The positive or negative signs indicate whether the modification underlies transcriptional silence or activity, respectively. Modifications of CENP-A required for its deposition or maintenance are also listed. See text for explanations. **(B)** Schematic outline of transcription-dependent inclusion of histone H3 variant CENP-A at the CEN chromatin, and recruitment of downstream kinetochore components as in vertebrates. See text for details.

Within the CEN domain, CENP-A nucleosomes are interspersed with canonical nucleosomes whose histone H3 tails are methylated at Lys4 (H3K4me1, H3K4me2) and Lys36 (H3K36me2, H3K36me3) (Figure 3A). These modifications underlie open chromatin, promote RNAPII activity, and are essential for HJURP targeting and CENP-A assembly (Bergmann et al., 2011; Duda et al., 2017). They also differentiate the CEN chromatin from the surrounding pericentromere regions, which are marked differently (see below and Figure 3A) (Sullivan and Karpen, 2004; Eymery et al., 2009; Gopalakrishnan et al., 2009; Bergmann et al., 2011, 2012). Intriguingly, H3K9me3, typically associated with transcriptional repression, also labels the centromeric nucleosomes (Bergmann et al., 2012) indicating that CEN chromatin epitomizes both silent heterochromatin and transcribed euchromatin (Sullivan and Karpen, 2004).

Histone H4 mono-acetylation at Lys5 and Lys12, which correlates with transcribed chromatin, is enriched at CEN and is essential for CENP-A deposition in chicken cells (Shang et al., 2016; Figure 3A). H4 mono-methylation at Lys20, which marks human and chicken CENP-A nucleosomes, and is associated with transcriptional activation, is a prerequisite for kinetochore assembly (Sullivan and Karpen, 2004; Vakoc et al., 2006; Wang et al., 2008; Bergmann et al., 2011; Hori et al., 2014). Histone H2B mono-ubiquitination at Lys119, catalyzed by the E3 ubiquitin ligase RNF20/40 (Brl1 in *S. pombe*), is required for CEN transcription (Zhu et al., 2011; Sadeghi et al., 2014). Depleting RNF20 reduces CEN transcription and nucleosome turnover, and causes chromosome missegregation in human cells and *S. pombe* (Sadeghi et al., 2014; Zhang et al., 2017). The ubiquitin ligase BRCA1 preserves CEN identity by ubiquitinating histone H2A at Lys119, producing a repressive mark. BRCA1 depletion, led to CEN transcript overexpression, impaired CEN cohesion and SAC activity, and chromosome missegregation (Di Paolo et al., 2014).

## Centromere Transcription, Promoters, and Transcription Factors

Although electron microscopy-based studies had localized RNA at kinetochores in union and salamander cells in the 1970s (Braselton, 1975; Rieder, 1979), CEN were long considered transcriptionally silent since they are confined in transcriptionally inert heterochromatin. Today we know that CEN are actively transcribed by RNAPII, which has been detected at CEN in *S. pombe*, flies, and human cells, at centromeric chromatin on human artificial chromosomes (HACs), and at neocentromeres (Wong et al., 2007; Li et al., 2008; Chueh et al., 2009; Ferri et al., 2009; Bergmann et al., 2011; Choi et al., 2011; Ohkuni and Kitagawa, 2011; Lyn Chan and Wong, 2012; Quénet and Dalal, 2014; Rošić et al., 2014; Catania et al., 2015). Despite the evidence of RNAII polymerase transcribing the CEN, very little is known about the promoters and transcription factors involved.

In *S. cerevisiae*, RNAPII-mediated CEN transcription is driven by transcription factors Cbf1 and Ste12. Cbf1 promotes transcription from the sense strand, Ste12 from the antisense strand. Silencing protein Dig1 inhibits Ste12. Transcriptional silencers Sir1, Hst1–Sum1, and Cdc14–Net1 associate with the CEN sequence, possibly to antagonize RNAPII. While deleting *CBF1* or *STE12* did not prevent kinetochore assembly, each mutant experienced chromosome loss. This phenotype was rescued by driving CEN transcription from an inducible promoter introduced next to the Cbf1- or Ste12-binding site, illustrating that CEN transcription is imperative for kinetochore activity (Ohkuni and Kitagawa, 2011). CEN transcripts in *S. cerevisiae* remained unidentified until exosome activity (which degrades non-coding RNAs) was removed, indicating a fast turn over of these transcripts. This approach revealed a 1.2-kb CEN3 RNA species, revealing that RNAPII proceeds into the pericentromere (Houseley et al., 2007). Low-level CEN transcription is required for kinetochore activity in budding yeast. Disproportionate CEN expression driven by the galactose-inducible *P*_GAL1_ promoter placed adjacent to CEN3 on a plasmid caused plasmid loss (Hill and Bloom, 1987) since kinetochores were not able to assemble. When *P*_GAL1_ was positioned next to chromosomal CEN3 that was marked with a GFP-array, growth in galactose prevented spindle binding of labeled sister chromatids 3. Following glucose addition, the sisters bi-oriented on the metaphase spindle (Tanaka et al., 2005).

In *S. pombe*, the CENP-A binding region contains numerous transcription start sites and promoters on the forward and reverse strands. However, very low levels of transcripts are produced, due to transcript turnover as well as RNAPII stalling (Choi et al., 2011; Sadeghi et al., 2014), which could result from collisions with the replisome or transient H2B (de)ubiquitination activity that negatively affects chromatin accessibility (Chen et al., 2008; Sadeghi et al., 2014). In fission yeast mutants unable to restart stalled RNAPII, CENP-A became actively deposited on the CEN, suggesting that halting RNAPII, which results in a low-quality transcription environment, allows for CEN chromatin remodeling and/or CENP-A loading (Shandilya et al., 2014; Catania et al., 2015).

The PRAT CEN satellite monomer in the beetle *Palorus ratzeburgii* contains a putative RNAPII promoter site that overlaps with the most conserved part of the PRAT sequence. This concurrence could be the result of selection pressure to preserve the transcription activity of this satellite DNA. TATA-box-like motifs, multiple transcription initiation and termination sites were also mapped within the monomer. The presence of a 5′-RNA cap and 3′-poly(A) tails in a portion of the beetle CEN transcripts indicates RNAPII-dependent transcription. Indeed, treatment of larvae with alpha-amanitin at concentrations that selectively inhibit RNAPII activity reduced the amount of PRAT transcripts. These transcripts derived from one, two, or three monomers, and were produced from both strands (albeit 10 times less from the antisense strand) (Pezer and Ugarković, 2008). Within the human alpha-satellite sequence, a candidate TATA box has been identified, as well as an SV40 enhancer-core sequence with spacing and orientation characteristic of RNAPII-transcribed genes (Vissel et al., 1992). In human cells, RNAPII has been found especially enriched at prometaphase, metaphase, and anaphase CEN, as well as at kinetochore-active neocentromeres. Consistent with active transcription, FCP1, a phosphatase that is specific for the carboxy-terminal domain of RNAP II and stimulates transcript elongation by RNAP II (Mandal et al., 2002), was identified at mitotic human and murine kinetochores (Chan et al., 2012).

## Transcription and Post-Translational Modifications Promote CENP-A Inclusion Into Centromeric Chromatin

While CENP-A represents the epigenetic mark of CEN identity in most eukaryotes (Vafa and Sullivan, 1997; Warburton et al., 1997) its presence *per se* is not enough for CEN formation since trypanosomes and insects with holocentric chromosomes lack a CENP-A ortholog (Akiyoshi and Gull, 2014; Drinnenberg et al., 2014). CENP-A nucleosomes in humans are also found at non-CEN sites, including neocentromeres (Bodor et al., 2014). Both observations underscore the need for additional CEN-specifying criteria, including structural themes embedded within the CEN DNA sequence (see above). Via its N- and C-terminal tails and through its central histone-fold domain, CENP-A recruits the other kinetochore proteins, including CENP-C with which it makes direct physical contact (Chen et al., 2000; van Hooser et al., 2001; Regnier et al., 2005; Liu et al., 2006; Carroll et al., 2009, 2010; Guse et al., 2011; Fachinetti et al., 2013; Kato et al., 2013; Folco et al., 2015; Logsdon et al., 2015; Westhorpe et al., 2015; Figure 3B). In contrast to histone H3, CENP-A may form a more rigid interface with its partner histone H4, which is further stabilized by CENP-C. Nucleosomes containing CENP-A bind less firmly to the DNA, profoundly affecting CEN transcription and distinghuishing it from the surrounding closed-state chromatin (Hasson et al., 2013; Falk et al., 2015). During chromosome replication, CENP-A becomes diluted 1:2 with histone H3 variant H3.3, which is deposited as a temporary placeholder allowing kinetochores to assemble in early metaphase (Figure 3B). In mammals, CENP-A becomes incorporated in late telophase/early G1, when its chaperone HJURP localizes to CEN and H3.3 is removed (Foltz et al., 2009; Dunleavy et al., 2011). CENP-A deposition also requires the MIS18 complex (MIS18α, MIS18β, MIS18-binding protein 1/KNL2) (Hayashi et al., 2004). In *D. melanogaster*, HJURP and MIS18 activities appear to be combined in the Cal1 protein (Erhardt et al., 2008; Chen et al., 2014).

In *S. cerevisiae*, the CEN nucleosomes are evicted and kinetochores disassembled at S-phase entry, allowing for the replication of the CEN sequences, which are the first loci to be replicated in budding yeast. It is unclear whether CEN transcription is downregulated during this process. The expelled CENP-A then becomes degraded. Within 5 min after passage of the replisome, the CEN nucleosomes reassemble by the inclusion of new CENP-A by the Scm3 chaperone (ortholog of HJURP). Kinetochores then reassemble to attach the still-replicating chromatids to the interphase spindle (Kitamura et al., 2007; Wisniewski et al., 2014).

During G1 in human cells, the MIS18 complex recruits the KAT7 histone acetyltransferase complex to maintain an acetylated CEN chromatin state, which facilitates the assembly of new CENP-A nucleosomes (Ohzeki et al., 2016). CENP-C contributes to CENP-A inclusion and stability by interacting directly with CENP-A, HJURP, and MIS18-binding protein 1 (Moree et al., 2011; Dambacher et al., 2012; McKinley and Cheeseman, 2014; Tachiwana et al., 2015; Figure 3B). Furthermore, CENP-C, the remodeling and spacing factor complex RSF, and the MgcRacGAP Male germ cell Rac GTPase-activating protein maintain CENP-A once incorporated (Perpelescu et al., 2009; Lagana et al., 2010; Falk et al., 2015). In contrast, cyclin-dependent kinase (CDK) activity negatively regulates CENP-A incorporation. In *D. melanogaster*, the turnover of S/G2 phase cyclin A in mitosis is key for the deposition of CENP-A (Erhardt et al., 2008; Mellone et al., 2011). In human cells, CDKs phosphorylate the MIS18-binding protein 1 to reduce its CEN localization (Silva et al., 2012) and to avert the recruitment of the MIS18α and MIS18β beyond G1 (McKinley and Cheeseman, 2014). CDK phosphorylation of HJURP also disrupts its CEN localization (Müller et al., 2014; Figure 3B). In contrast, the kinase PLK1 targets the MIS18 complex to promote its CEN localization and to license the CEN for CENP-A delivery. Bypassing both CDK and PLK1 activities led to CENP-A deposition throughout the cell cycle, causing severe mitotic defects (McKinley and Cheeseman, 2014). Clearly, CENP-A must be loaded only in G1 to ensure correct CEN function.

The *de novo* loading of CENP-A, as detailed above, requires CEN transcription as catalyzed by RNAPII (Lyn Chan and Wong, 2012; Quénet and Dalal, 2014; Rošić et al., 2014; Grenfell et al., 2016; Figure 3B). In *Drosophila*, Cal1 recruits RNAPII and the chromatin-remodeling complex FAcilitates Chromatin Transcription (FACT) (Foltz et al., 2006; Chen et al., 2015). Studies suggest that FACT activity weakens the histone core-DNA contact, facilitating the passage of RNAPII, and protecting the nucleosome from falling apart before it is remodeled and the new CENP-A nucleosome assembled. FACT also binds to the CEN CENP-T/W complex, possibly to promote also its deposition (Prendergast et al., 2016). Of note, FACT localizes at CEN at all stages of the cell cycle and is responsible for CENP-A loading in human cells (Okada et al., 2009). In fungi, FACT activity prevents the ectopic incorporation of CENP-A beyond CEN, rather than promoting CENP-A assembly at CEN nucleosomes (Deyter and Biggins, 2014). In *Drosophila*, CEN transcription and chromatin remodeling are required for CENP-A to transition from an unstable chromatin-associated state to a stable nucleosome-incorporated state (Bobkov et al., 2018).

Alpha-satellite arrays amplified from human CEN and cloned into a BAC plasmid form a functional HAC that recruits kinetochores and stably propagates in HT1080 fibrosarcoma cells (Maloney et al., 2012). In HACs containing engineered tetO operator sequences within the alpha-satellite DNA, and cells expressing transcriptional activators or silencers fused with the tetO-binding TetR protein both destabilized kinetochore formation (Bergmann et al., 2011). Transcriptional silencing led to a gradual loss of CENP-A from the centromeric chromatin, due to reduced recruitment of HJURP. Enhancing alpha-satellite transcription ∼10-fold by tethering a minimal NF-κB p65 activation domain did not affect kinetochore formation or activity. However, tethering TetR with the activation domain of herpes virus transcription factor VP16 elevated transcription ∼150-fold, approaching the expression level of a housekeeping gene. The consequent increase in RNAPII occupancy provoked a loss of CENP-A, probably through nucleosome eviction (Bergmann et al., 2012).

Post-translational modifications of CENP-A are required for its loading (Figure 3A). Before becoming deposited, CENP-A is phosphorylated at Ser16 and Ser18 (Bailey et al., 2013); Ser18 is a substrate for the cyclin E1/CDK2 kinase (Takada et al., 2017). A loss or hyperphosphorylation of both sites causes chromosome missegregation (Bailey et al., 2013; Takada et al., 2017). *Drosophila* CENP-A is phosphorylated at Ser75 and Ser77, which could be the analogs of Ser16 and Ser18 in human CENP-A (Boltengagen et al., 2016). Biochemical evidence suggests that mono-ubiquitination of CENP-A at Lys124 by the E3 ligase activity of the CUL4A–RBX1–COPS8 complex promotes HJURP binding and CENP-A deposition (Niikura et al., 2015, 2017). However, disputing gene replacement experiments showed that non-ubiquitinatable mutant CENP-A still can replace endogenous CENP-A and support cell viability (Fachinetti et al., 2017). In humans, the starting methionine of pre-inclusion CENP-A is removed and the exposed Gly1 residue trimethylated by the enzyme NRMT1 (Bailey et al., 2013; Sathyan et al., 2017). Both this modification and phosphorylation of Ser16 and Ser18 persist after CENP-A loading (Bailey et al., 2013). Subsequent modifications of the incorporated CENP-A include Ser7 phosphorylation, which is responsible for the indirect recruitment of CENP-C, and ubiquitination of Lys124, shown to be involved in CENP-A binding to HJURP (Srivastava et al., 2018). Mutations in Ser7, Ser16, and Ser18 sites lead to chromosome missegregation, abnormal spindles, and errors in cytokinesis (Srivastava et al., 2018). Nevertheless, chromosomes carrying CENP-A mutants that cannot be phosphorylated at Ser68 or ubiquitinated at Lys124 establish functional CEN (Fachinetti et al., 2017). Since the same amount of CENP-A is renewed at each G1 stage, errors in CENP-A incorporation caused by abnormal CEN transcription, assembly factor activity, and/or post-translational modifications could permanently alter its levels at centromeric chromatin, contributing to chromosomal instability.

## Centromere Transcription Through the Cell Cycle

Centromere transcription dynamics through the cell cycle have only been studied recently. The levels of alpha-satellite RNAs localizing at CEN did not change through the cell cycle, indicating a complex dynamic between CEN RNA synthesis, turnover, and stable incorporation in the CEN chromatin (McNulty et al., 2017). CEN RNA and DNA FISH experiments using identical HOR probes labeled with different fluorophores showed a co-localization of the transcripts to their originative CEN, indicating they are maintained in *cis* (McNulty et al., 2017). As discussed earlier, CEN transcription is required for CENP-A loading in human and *Drosophila* cells (Quénet and Dalal, 2014; Bobkov et al., 2018). Human CEN transcription mediated by RNAPII, in conjunction with the TATA-box binding protein, occurs through early G1 when mammalian CENP-A is deposited. When inhibiting transcription in G1, CENP-A levels dropped with ∼50% (Quénet and Dalal, 2014). Targeting the transcript with shRNA, while not impeding RNAPII activity, diminished CENP-A levels and induced mitotic defects (Quénet and Dalal, 2014). Reversely, depleting CENP-A reduced CENP-C concentrations at kinetochores, but CEN transcript levels were not affected, suggesting that CEN transcription occurs before the recruitment of CENP-A and CENP-C (McNulty et al., 2017). However, inhibiting active transcription resulted in CENP-C destabilization, suggesting that CEN transcription may also act downstream of CENP-A loading to promote CENP-C binding (Chan et al., 2012).

While most regions within condensed chromosomes are transcriptionally silent during mitosis, CEN are not (Chan et al., 2012; Lyn Chan and Wong, 2012; Liu et al., 2015), therewith differentiating them from the rest of the genome. Indeed, as indicated earlier, RNAPII localized at human and murine CEN from prometaphase through anaphase (Chan et al., 2012). Mild CEN transcription through the cell cycle ensures stable kinetochores and CEN cohesion (Liu et al., 2015).

In human cells, the cohesin-protecting protein Sgo1 (Shugoshin) is recruited to early mitotic kinetochores by the Bub1-phosphoryated centromeric histone H2A [phosphorylated at Thr120; (H2A T120P)]. Next, Sgo1 binds to RNAPII and travels along with it to the inner CEN (region between the two sister CEN) where it binds to the cohesin rings to protect them from precocious cleavage by the protease separase (Liu et al., 2015). Transcription by RNAPII and chromatin remodeling activities could open the chromatin, allowing Sgo1 access to cohesin. When transcription elongation was inhibited during mitosis with alpha-amanitin or when RNAPII subunit Rbp2 was degraded, Sgo1 still localized at kinetochores but did not relocate to the inner CEN. Besides RNAPII activity, the CEN RNAs themselves may facilitate Sgo1 relocation to the inner CEN. Indeed, since nonspecific RNA competed with H2A T120P for binding to Sgo1, CEN RNA could bind to Sgo1, releasing it from H2A T120P and allowing Sgo1 to travel with RNAPol II toward the inner CEN.

In contrast to human alpha-satellite transcripts, murine MinSat transcripts are absent in G0/G1. They appear in S-phase, peak at G2/M, and become undetectable after mitosis, when cells re-enter the cell cycle (Ferri et al., 2009). This dynamic mirrors the accumulation of the chromosomal passenger complex (CPC) at the murine CEN, implicating a role of MinSat RNAs in CPC localization and activity. Indeed, MinSat RNAs accumulate at CENP-A chromatin and interact with CPC subunits Aurora B and Survivin at mitotic onset. We will describe the interactions between CEN RNA and the CPC components in detail further below.

*Schizosaccharomyces pombe* CEN are transcribed during DNA replication, which may generate transcription–replication conflicts. Encounters between RNAPII and the replisome may cause RNAPII to halt and produce immature transcripts (Lu and Gilbert, 2007; Chen et al., 2008). RNAPII stalling generates RNA–ssDNA hybrids, known as R-loops (Reddy et al., 2011), which have also been observed at human CEN chromatin (Kabeche et al., 2018). R-loops must be resolved; otherwise, they can provoke chromosome breaks and repeat-sequence recombinations. R-loops forming in centromeric chromatin (or at pericentromeres or across the genome) trigger Aurora B-mediated phosphorylation of local histone H3 at Ser10, as shown in yeast, *C. elegans*, and human cells. This mark stimulates confined chromatin condensation and restricts DNA replication and transcription (Castellano-Pozo et al., 2013; Oestergaard and Lisby, 2016). Since the FACT complex resolves R-loops in yeast and human cells (Herrera-Moyano et al., 2014), it could remove toxic R-loops prior to mitotic entry. FACT activities including the stimulation of CEN chromatin remodeling and transcription, the subsequent promotion of CENP-A assembly, and the resolution of R-loops may reflect the dynamic state of the CEN environment during cell cycle progression (Duda et al., 2017).

## Post-Transcriptional Processing of Centromere Transcripts

In *S. pombe*, 5′-capped and 3′-polyadenylated non-coding CEN RNAs that are produced from the central domain are quickly degraded by the exosome (Choi et al., 2011). No evidence exists for small CEN RNA processing products as documented for the transcripts derived from the pericentromeric chromatin (see below). The RNase activity of exosome subunit Dis3 is required for correct kinetochore assembly and kinetochore–microtubule interactions (Bühler and Moazed, 2007; Mukarami et al., 2007) suggesting that degradation of CEN transcripts independent of the RNA interference (RNAi) pathway contributes to CEN activity in fission yeast.

Genome-wide screens with *Drosophila* and human cells identified splicing factors that are required for cell division (Goshima et al., 2007; Kittler et al., 2007; Somma et al., 2008; Neumann et al., 2010). Also, purifications of the spliceosome from HeLa cell nuclear extracts revealed the presence of microtubule- and mitotic chromatin-interacting proteins (Makarov et al., 2002). The processing of CEN RNAs may occur in mitosis since splicing factors are co-transcriptionally recruited to the elongating RNAPII transcripts (Listerman et al., 2006; David et al., 2011) (and because RNA-splicing factor Prp4 localizes to mitotic kinetochores in HeLa cells (Montembault et al., 2007; Figure 3B). Splicing factors also interact with MinSat transcripts in murine cells (Maison et al., 2011). The co-transcriptional recruitment of the RNA processing machinery to nascent mitotic transcripts in *Xenopus* is an important step in kinetochore and spindle assembly. Indeed, long non-coding CEN RNAs localize to mitotic chromosomes, chromatin, and spindles (Blower, 2016). At spindles, the transcripts regulate Aurora B and MCAK activities (Grenfell et al., 2016). Inhibiting the spliceosome, which co-IPs with CEN transcripts and CENP-C, in metaphase-arrested *Xenopus* egg extracts caused an accumulation of long CEN antisense transcripts representing up to six *frc1* monomer repeats, which are much longer than the standard CEN RNAs containing one to two *frc1* repeats. A globally reduced recruitment of CENP-A, CENP-C, and Ndc80 was observed (Grenfell et al., 2016), suggesting that *fcr1* antisense RNA is processed and then freely diffuses between CEN *in trans*, similar to observations in *Drosophila* where CEN RNAs derived from the X chromosome also move to the CEN of autosomal chromosomes (Rošić et al., 2014). However, the RNA signals appear not to have been completely removed from the autosomes after RNase treatment (Rošić et al., 2014) suggesting that FISH detected CEN DNA rather than the CEN RNA *in trans* (Bobkov et al., 2018).

In maize, CEN RNAs identified in IPs of CENP-A are produced from both strands and derived from the 156-bp CentC satellite monomer and transposable elements that are arranged in nearly continuous, intermingled arrays, and clusters. The transcripts are heterogeneous in length (40–200 nt) but predominantly contain 40 and 75-nt species (Du et al., 2010). Although these transcripts lie outside the range of microRNAs or siRNAs (20–30 nt) generated by RNAi pathways, their sizes indicate processing. The CEN RNAs are maintained in a single-stranded state within the maize kinetochore and are firmly bound to centromeric histone protein H3 (Topp et al., 2004), which may protect them from Dicer double-strand cleavage activity. Importantly, genuine siRNAs present in total RNA extracted from maize were not associated with CENP-A chromatin (Du et al., 2010).

Mouse embryonic stem (ES) cells knocked out in *dicer-1* (DCR^Δ/Δ^) are defective in global RNAi activity but retained ES cell characteristics. Although viable, they proliferated more slowly (Kanellopoulou et al., 2005). No aberrant chromosome structures or aneuploidy was observed but the cells displayed differentiation defects. The Dicer-negative cells contained increased levels of long, polyadenylated CEN MinSat, and pericentromere MajSat transcripts (>200 nt). Heterozygous mutant cells (DCR^Δ/+^) produced 150-nt MinSat and MajSat species, as well as 21–30 nt long specimen, suggesting the contribution of Dicer (Kanellopoulou et al., 2005). Further supporting the involvement of (peri)CEN RNA processing was the identification of protein WDHD1, which may stabilize the association of Dicer with MinSat and MajSat RNAs (Hsieh et al., 2011).

In tammar wallaby cells, 34–42 nt double-stranded (ds) RNAs with homology to the CEN retroelement kLTR (Ferreri et al., 2011) were identified in small-RNA pools (Carone et al., 2009; Lindsay et al., 2012). In rice, RNAs of ∼40 nt derive from the CentO CEN satellites (Lee et al., 2006). These rice and tammar wallaby CEN RNA species have been termed crasiRNAs (CEN repeat-associated short interacting RNAs). Targeting the small RNAs produced from the kLTR disrupted CENP-A localization in late telophase (Carone et al., 2009; Lindsay et al., 2012). Tight regulation and processing of these crasiRNAs seem integral to the epigenetic framework that is required for CEN establishment.

Hammerhead ribozyme structures associated with transcribed satellite DNA sequences have been identified in salamanders (Epstein and Gall, 1987), schistostome flatworms (Ferbeyre et al., 1998), and *Dolichopoda* cave crickets (Rojas et al., 2000). All hammerhead ribozymes self-cleave multimeric satellite transcripts into monomer RNAs.

## Centromere Proteins That Bind to Centromere Rna

Centromere transcripts or small CEN RNA derivatives underlie the formation of ribonucleoprotein complexes that specify the CEN domains and establish correct kinetochore assembly and architecture. These complexes comprise CENP-A, HJURP, CENP-B, CENP-C, the CPC, and Sgo1. While it is not clear how each protein interacts with the CEN transcripts, CENP-B, CENP-C, Sgo1, and the CPC have in common that their RNA-binding capacity serves as a second chromatin-recruitment mechanism. Indeed, it complements their promiscuous DNA binding activity (CENP-C), their binding to a specific satellite monomer box (CENP-B), their recruitment by the CEN H2A T120P modification (Sgo1), and their recruitment by Thr3-phosphorylated CEN histone H3 and CEN H2A T120P (CPC).

### CENP-A and HJURP

The interaction between CENP-A and CEN RNA was first observed at a human neocentromere. LINE-1 elements within the CENP-A-binding region of a neocentromere on 10q25 were transcribed into non-coding RNAs that integrate into the CENP-A chromatin (Chueh et al., 2009). Both CENP-A and HJURP interact with CEN RNA as shown in alpha-satellite transcript pull-down experiments (Quénet and Dalal, 2014). *In silico* predictions of potential RNA-binding sites indicated that 286 out of the 748 HJURP residues, and 79 out of the 140 CENP-A residues, have RNA-binding capacity. However, the vast majority of these CENP-A residues may well be buried inside the nucleosome and/or be bound by CENP-C and CENP-N. The predicted residues lie in the N-terminal half of CENP-A, the protein’s most rapidly evolving part (Henikoff et al., 2001; Malik and Henikoff, 2001), which is required to stabilize CENP-A at centromeric nucleosomes (Logsdon et al., 2015). Possibly, the disparities in composition and length of the N-terminal halves of all CENP-A orthologs could allow for their interaction with the rapidly evolving CEN DNA and, consequently, CEN RNA sequences.

### CENP-C

CENP-C, which acts as a dimer via its C-terminal dimerization domain (Cohen et al., 2008), binds to CENP-A, CEN DNA, and RNA (Figure 3B). Single-stranded alpha-satellite RNA localizes CENP-C to CEN in interphase, which then together with CENP-A recruits the other kinetochore proteins. Two regions in human CENP-C (one central and one C-terminal) preferably bind to CEN RNAs as shown in competition assays with rRNA, tRNA, and murine MajSat RNA (Wong et al., 2007). However, both sequences also bind to CEN DNA (Sugimoto et al., 1997; Yang et al., 1996). Each DNA-binding element contains a 21–22 amino acid motif via which CENP-C also contacts CENP-A (Kato et al., 2013). Mutating three lysine residues adjacent to CENP-A’s central DNA-binding motif also abrogated RNA binding in that region (Wong et al., 2007). Noteworthy, CENP-C’s central RNA-binding domain shares homology with the RNA-binding hinge domain region of the pericentromeric heterochromatin proteins (HP) HP1α, β, and γ (Du et al., 2010; Muchardt et al., 2002).

In maize, a C-terminal 122-residue CENP-C region encoded by exons 9–12 binds RNA and DNA, and is required for its CEN localization *in vivo*. While maize CENP-C binding to CEN RNA occurs without any sequence specificity (in contrast to human CENP-C), CEN DNA binding is stabilized by long ssRNA *in vitro*. The RNAs that stabilize this contact correspond to the ssCEN RNAs present in kinetochores (Du et al., 2010). Possibly, CEN ssRNA may stabilize CENP-C by enhancing its binding to CEN DNA, adjacent to where it interacts with the CENP-A nucleosome. Indeed, disrupting CEN RNA destabilizes CENP-C at the CEN. Treating mitotic human cells with alpha-amanitin lowered CENP-C levels at kinetochores and caused an increase in lagging chromosomes. A relatively greater reduction of CENP-C occurred on the lagging chromosomes compared to the chromosomes that segregated (Lyn Chan and Wong, 2012). Impeding transcription initiation or splicing also led to decreased CENP-C levels at kinetochores in *Xenopus* (Grenfell et al., 2016). In *Drosophila*, X chromosome-specific SatIII transcripts localize to CEN and associate with CENP-C (Rošić et al., 2014). Following CENP-C depletion, the SatIII RNA signals at CEN dropped. Reversely, when depleting SatIII RNAs, the presence of newly synthesized CENP-C and CENP-A at CEN was reduced. This negative effect cascaded up through the kinetochore (Rošić et al., 2014). Taken together, results with human cells, *Drosophila*, maize, and *Xenopus* suggest that the non-coding CEN RNAs recruit and stabilize CENP-C, supporting CENP-A deposition and stability.

CENP-C bound to CEN DNA and RNA also interacts with chromatin modifying proteins to create the unique epigenetic environment of the CEN domain. CENP-C recruits DNA methyltransferase 3A-B (DNMT3A-B) to reduce local transcription by promoting the methylation of CEN DNA and histone H3. Consequently, CENP-C depletion caused increased CEN transcription (Gopalakrishnan et al., 2009). CENP-C also binds to MIS18 complex components MIS18α- and MIS18-binding protein 1 (Moree et al., 2011; Kim et al., 2012), which control CEN histone acetylation (Fujita et al., 2007). Mis18α through its interaction with DNMT3A-B can also control DNA methylation and histone modifications (Kim et al., 2012), whereas CENP-C through its interaction with M18BP1 promotes the recruitment of HJURP for CENP-A loading (Moree et al., 2011). Possibly, CEN RNA stabilizes CENP-C:DNMT3A-B:MIS18 to target HJURP:CENP-A.

### The Chromosomal Passenger Complex

The binding of CENP-A and CENP-C to CEN DNA and alpha-satellite RNA promotes kinetochore assembly, including the recruitment of the 4-protein CPC (INCENP, Survivin, Borealin, and Aurora B), which regulates chromosome-spindle attachment and activates the SAC upon chromosome misalignment (Hindriksen et al., 2017). The CPC moves from the inner CEN to the spindle midzone in late anaphase to regulate cytokinesis (Warecki and Sullivan, 2018). Aurora B also phosphorylates CENP-A at Ser7 (Zeitlin et al., 2001). Both proteins coincide at the CEN in metaphase and move to the contractile ring in cytokinesis. Possibly, CEN RNA acts as a scaffold to promote their re-localization.

Knocking down alpha-satellite RNA in human cells (Ideue et al., 2014) or inhibiting transcription in *Xenopus* egg extracts (Blower, 2016) reduced the CEN levels of Aurora B, resulting in unaligned chromosomes caused by improper spindle attachment. Overexpressing MinSat RNA equally mislocalized Aurora B in murine cells, instigating chromosome misalignment and aneuploidy (Bouzinba-Segard et al., 2006). Moreover, Aurora B kinase activity was regulated by MinSat RNA levels (Ferri et al., 2009). Nonetheless, ectopic overexpression of satellite I RNA did not significantly affect chromosome segregation and CEN functions in human cells (Ideue et al., 2014).

The RNA-dependent inner kinetochore localization of the CPC is mediated by at least two RNA-binding domains: one that is present in Aurora B and one in Survivin or Borealin (Blower, 2016). Aurora B and recombinant CPC also bind to RNA *in vitro*. RNA stimulates Aurora B kinase activity *in vitro* and *in vivo*, and a positive feedback loop exists between its kinase activity and its metaphase localization (Wang et al., 2011; Jambhekar et al., 2014). CPC assembly and Aurora B activity were sensitive to RNase treatment. However, kinase activity was rescued with RNA, perhaps via allosteric effects on Aurora B binding (Ferri et al., 2009; Ideue et al., 2014; Jambhekar et al., 2014). Pull-downs of MinSat RNA from murine cells recovered CENP-A, Aurora B, Survivin, and INCENP (Ferri et al., 2009). Reciprocally, CEN RNAs of murine and human cells co-immunoprecipitated with CENP-A, Aurora B, Survivin, and INCENP (Ferri et al., 2009; Ideue et al., 2014).

Besides CEN RNA, *Xenopus* Aurora B also interacts with other RNAs (including mRNAs) to form ribonucleoprotein complexes, as observed in anti-Aurora B immunoprecipitation experiments with interphase and mitotic cells, followed by RNA-sequencing. Over 600 RNAs were identified, 465 of which were specific for mitosis, suggesting a cell cycle-regulated binding of target RNA. Identified RNAs encode proteins of the cytoskeleton, centrosome, transcription factors, and RNAs that are enriched on spindle microtubules (Jambhekar et al., 2014). While the RNA pool showed an overrepresentation of adenines, Aurora B interacted rather promiscuously with RNA, and bound *in vitro* only with minor preference to the *Xenopus fcr1* CEN satellite transcript (Blower, 2016).

## Heterochromatic Pericentromeres Insulate the Centromere

Centromeric chromatin in fission yeast and metazoans is flanked by constitutive heterochromatin. The pericentromeric domains bind specific proteins and carry epigenetic marks that keep them in a transcriptionally inert state thereby insulating themselves from the enclosed CEN. Pericentric chromatin stabilizes the CEN domain by preventing internal recombinations between intra-CEN repeat sequences (Hetrr and Allis, 2005). It also actively recruits cohesin (via the SUV4-20H2 methyltransferase enzymes that trimethylate histone H4 at Lys20) to promote the bi-orientation of and tension development between the sister chromatids (Bernard et al., 2001; Sakuno et al., 2009; Yamagishi et al., 2010; Yi et al., 2018).

Similar to the CEN sequence, pericentromeres comprise simple repeat sequences such as alpha-satellite DNA, beta-, gamma-, I, II, and III satellite sequences (5–200 bp). They further contain DNA transposons (1 kb), long terminal repeat (LTR)-endogenous retroviral elements (10 kb), non-LTR autonomous retrotransposons (transposons that are formed after reverse transcription of an intermediate RNAPIII-generated transcript) including long interspersed elements (LINEs, 6 kb) and short interspersed elements SINE (100–300 bp) (Figure 2C). Pericentromeres harbor promoter elements that recruit various transcription factors, including Ikaros in human cells (Gurel et al., 2008), the ubiquitous YY1 at murine gamma-satellites (Shestakova et al., 2004), Nanog and Sall1 in mouse ES cells (Lopes Novo and Rugg-Gunn, 2016) to regulate transcription by RNAPII or RNAPIII (Pezer and Ugarković, 2008). The repeat sequences are not conserved between or within a species, suggesting that pericentromere transcription is epigenetically controlled. Indeed, it contains histone H3 variants H3.3 and H2A.Z (Drané et al., 2010; Santenard et al., 2010) and binds the conserved HP1, which propagates the heterochromatic state and coordinates chromatin silencing, cohesion, and replication activities (Saksouk et al., 2015). The pericentric histones are hypoacetylated, resulting in chromatin fiber compaction. Methylation marks are enriched on histone H3; H3K9me2, H3K9me3 (recognized by HP1), H3K27me2, and H3K27me3, but also on histone H4; H4K20me2, H4K20Me3, and on cytosine and adenine (Gopalakrishnan et al., 2009; Rose and Klose, 2014; Figure 3A). Notwithstanding this repressive environment, pericentromeres are transcribed in many organisms. A delicate balance between pericentromere and CEN transcription ensures chromosomal stability (see next).

## Pericentromere Transcription and Transcript Processing Ensure Its Silent State

**FIGURE 4:**
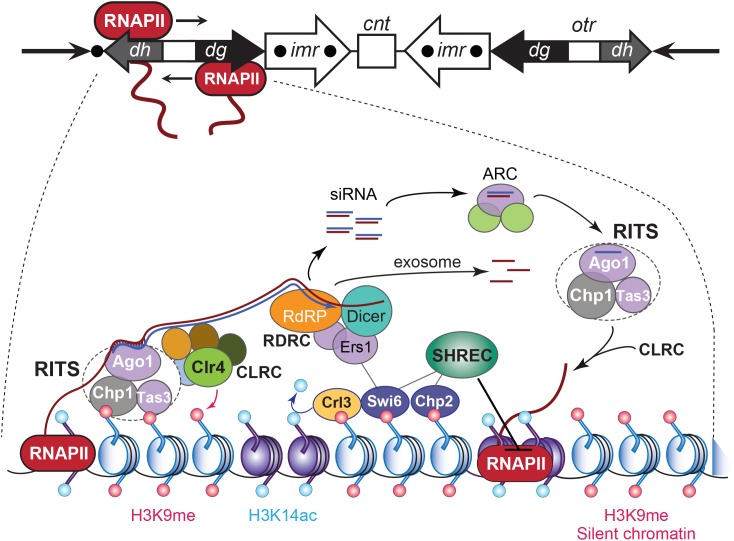
RNA interference-based heterochromatin formation and maintenance at *S. pombe* CEN. Upper panel: the *S. pombe* CEN 1, indicating transcription by RNAPII of an outer repeat *otr* element that flanks the central core of the CEN. Lowe panel: Regulation of the heterochromatic state of CEN sequences that flank the central core domain. The RNA-induced transcriptional silencing (RITS) complex binds to ssRNA transcripts generated from the *otr* sequence repeats, by siRNA–RNA base pairing interactions and via nucleosomes by localizing to histone H3 methylated at Lys9 (H3K9me). RITS then recruits RDRC/Dicer activity, promotes dsRNA synthesis, the production of siRNAs, and CLRC H3K9 methyltransferase-mediated H3K9 methylation. The Argonaute siRNA chaperone complex (ARC) catalyzes the transfer of the siRNAs from RDRC/Dicer to the RITS complex. The transcript ssRNAs present in the siRNAs become degraded by the exosome. Chromodomain HP1 proteins Swi6 and Chp2 are recruited by the H3K9me mark and silence transcription of the chromatin by localizing the chromatin remodeling Snf2/HDAC repressive complex (SHREC), which inhibits RNAPII activity. Adapted from Holoch and Moazed (2015).

In *S. pombe*, repressive H3K9 methylation occurs at the outermost *dg* and *dh* pericentromere repeats and ends at the tRNA clusters inside the innermost repeats that surround the CEN’s central domain. Their presence prevents the pericentromeric heterochromatin from expanding into the CENP-A chromatin (Cam et al., 2005; Figure 4). The tRNA clusters are transcribed by RNAPIII, which further delineates the CEN core domain from the flanking pericentromeres (Partridge et al., 2000; Scott et al., 2006). RNAPIII barrier transcription activity does not depend on the orientation of the tRNA genes, but on the DNA sequence that is required for formation of the RNAPIII complex (Scott et al., 2006, 2007). The retrotransposon SINE, found throughout the mammalian genome, is also transcribed by RNAPIII at pericentromeres. SINE expression has been linked to establishing boundary elements and chromatin insulators across the genome (Lunyak et al., 2007; Román et al., 2011). Similarly, SINE transcription and/or that of other pericentric DNA elements could insulate the CEN from the bulk chromatin.

Transcription of pericentromeric chromatin occurs in many species and, except for the tRNA genes in fission yeast, is largely devoid of protein-encoding sequences (Brown et al., 2012; Hall et al., 2012; Saksouk et al., 2015). In *S. pombe*, small-interfering RNAs (siRNAs) produced after the processing of longer transcripts are required for the propagation and maintenance of the heterochromatic identity of pericentromers (Volpe et al., 2002). The finding that transcription of pericentromeric chromatin is functionally significant led to a re-assessment of the definition of “silent” heterochromatin. Specifically, RNAPII bi-directionally produces pre-RNAs from cryptic and TATA-like promoter sequences within the *dh* and *dg* elements of the *otr* regions that border the central CEN (Reinhart and Bartel, 2002; Djupedal et al., 2005; Kato et al., 2005; Figure 4). Both *otr* elements are not required for CEN function during mitosis but provide a platform for the heterochromatin component of *S. pombe* CEN (Kagansky et al., 2009). The produced single-stranded polyadenylated transcripts are converted into dsRNA species by the RNA-directed RNA polymerase-containing RDRC complex, which Dicer (Dcr1) next processes into short siRNAs that are transferred by ARC (Argonaute siRNA chaperone complex) to the Argonaute (Ago1)-containing RNA-induced transcriptional silencing complex RITS (Volpe et al., 2002; Martienssen et al., 2005). Through their interaction with Ago1, the siRNAs load RITS onto the cognate pericentromeric chromatin via base-pairing with the nascent transcripts. The RITS complex then recruits the CLRC complex that contains the histone methyltransferase Clr4 (SUV39H in mammals), which methylates H3K9. The latter recruits chromodomain proteins Swi6 (*S. pombe* HP1 ortholog) and the SHREK-associated protein Chp2, as well as histone deacetylase Clr3 (HDAC1), which removes the local permissive H3K14ac marks. The SHREK complex inhibits RNAPII activity, resulting in silent heterochromatin (Figure 4).

Deleting RNAi pathway genes (*dcr1*, *ago1* or *RdP1*) caused chromosome missegregation due to defective silencing of the pericentromeric heterochromatin. *S. pombe* strains mutated in RNAPII subunits Rpb2 and Rpb7 also suffered from increased chromosomal instability, impaired transcriptional silencing, and a reduced association of H3K9me and Swi6 at *dg*/*dh* (Djupedal et al., 2005; Kato et al., 2005). Pericentromere transcription and siRNA production in *S. pombe* peak in S-phase. Hence, pericentromere silencing may be alleviated in S-phase as heterochromatin markers H3K9me and Swi6 become distributed on the replicated strands (Chen et al., 2008; Kloc et al., 2008). Without RNAi, homologous recombination repairs the stalled forks (Zaratiegui et al., 2011) suggesting that transcriptional silencing of pericentromeric heterochromatin prevents replication stress (Castel and Martienssen, 2013).

The importance of Dicer-dependent processing of pericentromere RNAs for heterochromatin assembly in vertebrates was demonstrated with chicken DT40 cells carrying a human chromosome (Fukagawa et al., 2004). Eliminating Dicer provoked an accumulation of long pericentric alpha-satellite and SatIII transcripts, and caused mitotic defects due to precocious sister chromatid separation; attributed to HP1 loss and a misregulation of cohesin and SAC protein BubR1. Similarly, conditionally depleting Dicer in mouse ES cells led to an accumulation of short MajSat transcripts (40 to >200 nt) and the normally repressed long interspersed repeated DNA and high-copy-number LTR retrotransposons. These findings indicated a role for Dicer in repressing pericentromere regions and other usually silent genetic elements (Kanellopoulou et al., 2005). Since the binding of HP1 to heterochromatin requires RNA (Maison et al., 2002; Muchardt et al., 2002), the Dicer-processed siRNAs were assumed to represent them. However, other than in chicken cells (Fukagawa et al., 2004), 21–25 nt siRNAs deriving from the pericentromeric domains have been difficult to identify in vertebrates. Irrespective of how or if the RNAi pathway contributes, pericentromere transcripts in mammals seem involved in the formation and maintenance of heterochromatin. For example in mice, protein WDHD1, which plays a role in RNAPII transcription and RNA processing, interacts with MajSat transcripts. Depleting WDHD1 enhanced MajSat levels and reduced pericentromeric heterochromatin condensation, resulting in proliferation defects (Hsieh et al., 2011). Additional work with mouse early embryos showed that injections of satellite dsRNAs can localize HP1β to pericentromeres revealing that HP1 is targeted in an RNA-dependent, sequence-specific manner. However, a functional association with the RNAi machinery was not assessed (Santenard et al., 2010).

Long non-coding transcripts corresponding to several MajSat satellite repeat units specifically associate with SUMOylated HP1, which is stabilized by H3K9me3, in murine cells. RNase treatment released HP1 and altered the spacing of the pericentromeric histones. HP1 preferentially binds to the forward strand of these RNAs, which remains bound to the site of transcription. Additional HP1 molecules then accumulate, connecting pericentromere transcription with heterochromatin formation (Maison et al., 2011). In primary mouse embryonic fibroblasts, pericentromeric heterochromatin transcription is proliferation- and cell cycle-dependent (Lu and Gilbert, 2007). A first pool of long, heterogeneous MajSat transcripts (1 kb to >8 kb) is produced by RNAPII through G1 and peaks in G1/S-phase, right before pericentromere replication (mid-to-late S-phase). Since the transcripts accumulate at the site of pericentromere replication, local transcription could promote heterochromatin reassembly at the replication fork. A pool of shorter transcripts (∼200 nt) is produced at mitotic onset, coinciding with transcription factors and other proteins becoming cleared from the heterochromatin. This transcript population/transcription activity could be involved in heterochromatin formation, maintenance, and reinforcement during the later stages of mitosis when cohesin at pericentromeres has been removed (Wu et al., 2006). Indeed, while HP1 is dispatched from heterochromatin during M-phase (Muchardt et al., 2002; Fischle et al., 2005), H3K9me3 and the short M-phase RNAs could contribute to the anaphase recruitment of HP1 (Saksouk et al., 2015). SUV39 (Suv39h) histone lysine methyltransferase promotes constitutive heterochromatin compaction and transcriptional repression by catalyzing the H3K9me2/3 modification in humans and mice. SUV39 is incorporated and stabilized in constitutive heterochromatin by chromatin-associated non-coding RNAs (Johnson et al., 2017; Velazquez Camacho et al., 2017).

Heterochromatin activity in *D. melanogaster* is also associated with histone H3K9 methylation by Su(var)3-9 and HP1 recruitment (Ebert et al., 2006). Involvement of siRNA pathways acting in heterochromatin formation in *Drosophila* has been hypothesized since a nuclear pool of transposable element-derived siRNAs (21 nt) was shown to promote heterochromatin formation in somatic cells of *Drosophila.* Components of the RNAi pathway contributed to heterochromatin maintenance (Fagegaltier et al., 2009). As in *S. pombe* and mammals, these siRNAs might tether complementary nascent transcripts of satellite DNAs and transposons, and guide chromatin-modifying enzymes, including Su(var)3-9. RNAi activity seems to help establish heterochromatin in the early embryo, which can then be maintained in the absence of RNAi in somatic tissues (Huisinga and Elgin, 2009). Contrary to *D. melanogaster*, plants often contain a significant portion of methylated repetitive DNA. In fact, siRNAs guiding the methylation of histones and DNA at the loci they were derived from (Zakrzewski et al., 2011). Processing of satellite-derived transcripts by the RNAi pathway into siRNAs (21–24 nt) has been reported for *Arabidopsis*, rice, and sugar beet (May et al., 2005; Lee et al., 2006; Zakrzewski et al., 2011). Small RNAs with a predominant size of 24 nt cognate to the satellite TCAST (Ugarković et al., 1996; Feliciello et al., 2011) have been detected in the beetle *Tribolium castaneum* and are more abundant in embryos than in later developmental stages (Pezer and Ugarković, 2008; Pezer et al., 2012). The sequences of components of the RNAi pathway are present in the genome of *T. castaneum*, including Argonaute and Dicer, but not the RNA-dependent RNA polymerase gene (Tomoyasu et al., 2008), which insects and vertebrates appear to lack.

## Centromere and Pericentromere Transcription During Development and Differentiation

Satellite DNA has been associated with differentiation and development. Repetitive DNA is not transcribed in adult tissues presumably because it is hypermethylated (Jeanpierre et al., 1993) while it is hypomethylated in fetal tissues (Miniou et al., 1997). Antisense MajSat transcripts accumulate in the central nervous system of mouse embryos 11.5 days post coitum (dpc), and become replaced by sense MajSat transcripts from 12.5 until 15.5 dpc. In adult mice, MajSat transcripts were identified only in highly proliferative tissues such as liver and testis (Rudert et al., 1995). In chicken and zebrafish, alpha-satellite expression from the sense and antisense strands occurs in a regulated pattern during embryogenesis, possibly to control gene expression following transcript processing (Li and Kirby, 2003). Before headfold formation in the chick and at 0–2 h post-fertilization (hpf) in zebrafish, blastodiscs expressed the alpha-repeat sequences. By stage 9 and at 6–8 hpf, respectively, the expression localized to the head mesoderm, myocardium, pharyngeal endoderm, and cardiac neural crest. Because the expression occurred so early in zebrafish, the authors looked for the alpha-repeat transcripts within the maternal RNAs in single-cell and four-cell stage embryos. These stages occur within minutes of fertilization and before the start of zygotic transcription at 3 hpf. High levels of the transcripts were found, supporting their maternal origin (Li and Kirby, 2003).

## Anomalous Centromere and Pericentromere Transcription During Stress and Disease

Since the centromeric and pericentromeric regions are epigenetically controlled, any loss/reduction in repressive marks such as DNA and histone methylation or increased removal of active acetylation marks can provoke satellite overexpression from the centromeric and pericentromeric regions as observed during stress, senescence, aging, and in cancer cells. Pathological transcription of either region dramatically affects CEN insulation and activity, resulting in disturbed kinetochore formation and genetic instability.

### (Peri)centromere Transcription During Stress

**FIGURE 5:**
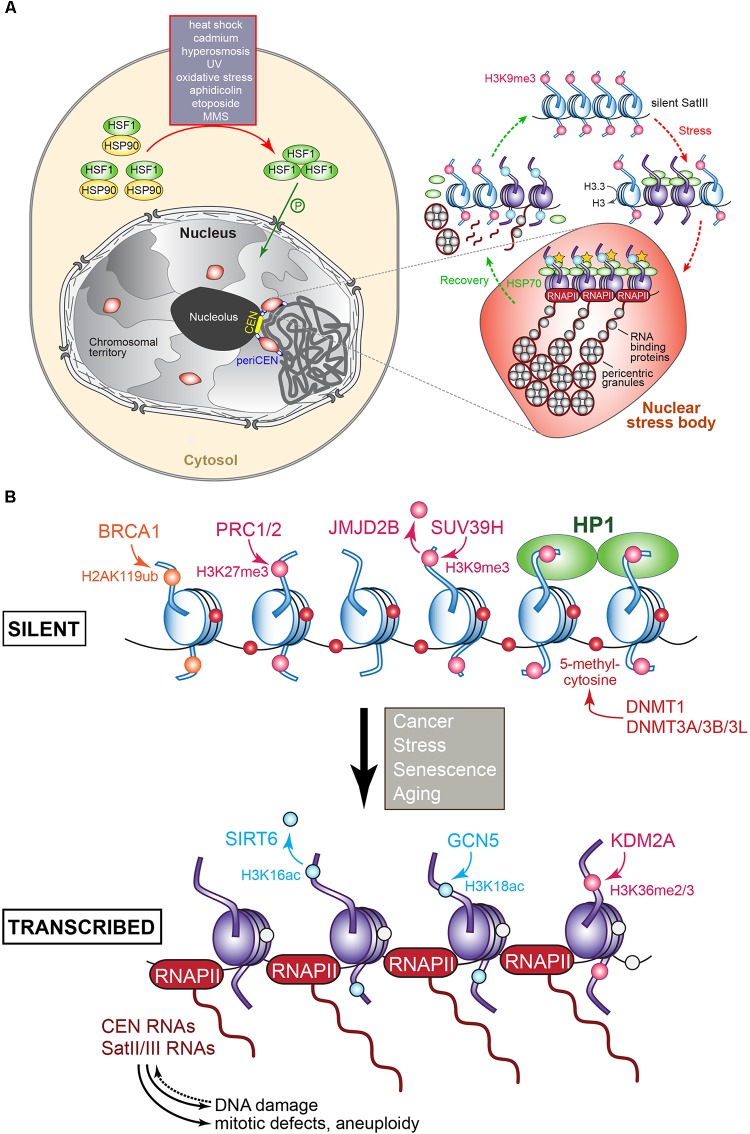
**(A)** Regulation of pericentromere SatIII transcription in human cells following exposure to heat and other stresses. In the absence of stress, SatIII repeat sequences are epigenetically marked for silence (H3K9me, pink dot) and exist in a closed transcriptionally inert state (blue nucleosomes). Upon exposure to heat or other stresses, the monomeric HSF1 (shown in green) becomes upregulated, and forms homotrimers that after phosphorylation enter the nucleus. The HSF1 bind to the SatIII sequences and recruit the histone acetyltransferase (HAT) CREB-binding proteins to trigger histone hyperacetylation (yellow stars), which results in active SatIII transcription by RNAPII of one strand. A subset of RNA-binding/processing proteins is recruited to the SatIII transcripts, forming ribonucleoprotein complexes that associate into so-called perichromatin granules, which in turn produce clusters that correspond to a mature nuclear stress body (represented by the red oval structure). To recover from the inflicted stress, heat shock protein HSP70 induces the disassembly of the HSF1 trimers that leave the nuclear stress bodies, along with RNAPII and the HAT. The granules disassemble and the RNA-binding proteins redistribute throughout the nucleoplasm. SatIII transcripts may become processed into smaller fragments possibly by the RNAi machinery to protect and re-establish the heterochromatic state of the pericentromeric region comprising the SatIII repeats, possibly by recruiting epigenetic writing activity resulting in the establishment of the repressive H3K9me signals. Adapted from Biamonti and Vourc’h (2010) and Biamonti (2004). **(B)** Transcriptional regulation of (peri)centric repeat sequences as identified in various tumors. The epigenetic marks and the enzymes responsible for introducing or removing them at histones or cytosine are indicated. Blue nucleosomes: silent, purple nucleosomes: transcribed. The overproduction of (peri)centric transcripts can induce DNA damage, mitotic defects, genomic instability, and aneuploidy. See text for details.

In human cells, the transcription of certain pericentromeric satellite sequences, in particular SatIII, is induced upon heat shock and exposure to heavy metals, chemicals, UV radiation, hyperosmotic, or oxidative conditions (Figure 5A). Importantly, while SatIII transcripts were up-regulated following heat shock, CEN transcripts were not, indicating that each domain is subject to different transcriptional control mechanisms (Jolly et al., 2004; Rizzi et al., 2004; Valgardsdottir et al., 2008; Eymery et al., 2009). SatIII expression levels also depend on the type of stress that is experienced: MMS, etoposide, aphidicolin, and oxidative stress are weak inducers; UV and hyperosmosis have a moderate effect; and heat shock and cadmium are very strong activators. In unstressed cells, SatIII sequences exist in a transcriptionally silent, closed heterochromatin conformation. Following heat shock or stress, SatIII transcription is induced (Valgardsdottir et al., 2008). Specifically, monomeric transcription factor Heat Shock transcription Factor 1 (HSF1) becomes upregulated and binds as a phosphorylated homotrimer to the SatIII sequences. HSF1 then recruits the histone acetylase CREB-binding protein CBP to trigger histone hyperacetylation while the death domain-associated protein DAXX, which acts as a chaperone for pericentromeric histone H3.3, promotes SatIII transcription by RNAPII. Upon DAXX depletion, SatIII expression levels in heat-shocked cells dropped, while less H3.3 was incorporated (Morozov et al., 2012). A set of RNA-binding and processing proteins associate with the SatIII transcripts. RNAi knock-downs of these transcripts that range between 2 and 5 kb (Jolly et al., 2004; Rizzi et al., 2004) reduced the recruitment of RNA processing factors, including the splicing factor SF2/ASF (Chiodi et al., 2004; Metz et al., 2004). The RNA-binding factors and SatIII transcripts produce ribonucleoprotein complexes that combine into many perichromatin granules. Together, they correspond to mature nuclear stress bodies that accumulate at the pericentromeres (Denegri et al., 2002; Jolly et al., 2004; Figure 5A). The number and size of the nuclear stress bodies correlate directly with SatIII expression (Valgardsdottir et al., 2008). During recovery from the stress, increased levels of heat shock protein HSP70 trigger the disassembly of the HSF1 trimers, which leave the nuclear stress bodies together with the histone acetyltransferase CBP and RNAPII. Next, the granule clusters dissociate, the RNA-binding proteins redistribute through the nucleoplasm but the SatIII transcripts stay bound to the granules. At the same time, granules that are H3K9 methylated appear adjacent to the disassembling nuclear stress bodies. The transcripts are then cleaved, and a complex similar to the *S. pombe* RITS complex may then localize the transcripts to the chromatin to silence the SatIII DNA arrays (Biamonti, 2004; Biamonti and Vourc’h, 2010; Figure 5A). Depending on the stress that is experienced, different transcription factors promote SatIII activation. For example, the tonicity-responsive enhancer binding protein TONEBP induces SatIII expression under hyperosmotic stress (Valgardsdottir et al., 2008). Satellite transcript accumulation during heat stress also occurs in insects (Pezer et al., 2012) and plants (Tittel-Elmer et al., 2010). In the beetle *T. castaneum* pericentromere TCAST satellites are transcribed by RNAPII and processed into 21–30 nt siRNAs. The production of these siRNAs is developmentally regulated but is strongly induced upon heat shock. During recovery, siRNA expression and histone modifications are restored to normal. Transient heterochromatin remodeling seems part of a stress-activated gene-expression program in beetles (Pezer et al., 2012), and possibly other organisms as well. In *Arabidopsis*, a temperature upshift alleviated the silent state of CEN satellite sequences, pericentric 5S rDNA arrays, transposable elements, and 106B interspersed repeats. Surprisingly, the pattern of repressive epigenetic marks within the heterochromatin was not affected, suggesting that the temperature-stimulated transcription activity bypassed these regulatory modifications (Tittel-Elmer et al., 2010).

Centromere MinSat transcription increases when murine cells are exposed to chemical stress (DMSO, 5-aza-2′-deoxycytidine, apoptosis inducer staurosporine). Comparable ectopic overexpression of MinSat DNA led to decondensed CEN and mitotic defects such as multiple spindle attachments, loss of sister chromatid cohesion, aneuploidy, and cell death (Bouzinba-Segard et al., 2006).

Extensive evidence points to an interplay between the DNA damage response and satellite DNA expression. Ectopic expression of satellite RNA in cultured human mammary epithelial cells induced numerous foci of γ-H2A.X, the phosphorylated histone H2A.X variant that marks dsDNA breaks (Zhu et al., 2011). These cells also exhibited bridged and lagging chromosomes as well as disorganized metaphase spindles (Zhu et al., 2018). Similarly, MajSat RNA overexpression compromised DNA damage repair, resulting in high DNA mutation rates in cultured murine pancreatic cells (Kishikawa et al., 2016a, 2018). Elevated levels of γ-H2A.X were also observed after nuclear injection of satellite RNA in human cells, indicating that high transcription intensity *per se* does not trigger the DNA damage response (Zhu et al., 2018). CRISPR-mediated activation of MinSat and MajSat expression in murine cells incited chromosomal instability (Zhu et al., 2018). Vice versa, genotoxic etoposide treatment (causes dsDNA breaks) triggered MinSat transcription and CENP-A eviction from the mouse CEN, which relied on the p53-dependent DNA damage pathway and chromatin chaperone/remodeling factors (Hédouin et al., 2017). In the absence of functional p53, DNA demethylation as induced by 5-aza-2′-deoxycytidine resulted in massive transcription of MajSat RNA in mouse fibroblasts (Leonova et al., 2013). Moreover, ectopic overexpression of MajSat RNA stimulated by injection of sh-p53 RNA causing p53 knockdown led to tumor formation (Zhu et al., 2018).

How do (peri)centromere transcription/transcripts contribute to a stress response and recovery from it? The transcripts processed via an RNAi-dependent or -independent pathway could mediate heterochromatin reformation, as in *S. pombe*. Analogous to X-chromosome inactivation by the long non-coding Xist RNA, the transcripts might recruit chromatin remodelers and DNA methyltransferases to establish a silent pericentric state. Also, SatIII transcripts could protect a fragile region of the genome from stress-induced DNA damage (the SatIII-enriched 9q12 region is often rearranged in pathologies, including cancer). Possibly, the transcripts could regulate local RNA splicing during the stress response by sequestering splicing factors. Via a position-effect mechanism, they might counteract the repressive nature of heterochromatin and activate nearby genes in *cis* or *trans* (Eymery et al., 2009; Saksouk et al., 2015).

### (Peri)centromere Transcription During Senescence and Aging

Heterochromatin structure and expression change during aging. An up-regulation of MajSat expression in senescent cardiac muscle cells of aging mice but not in their brain or kidneys may be linked to mitochondria-induced oxidative stress (Gaubatz and Cutler, 1990). Transcriptional activation of pericentromeres has been observed in replicative senescence and aging. Upon extensive passaging of human fibroblasts, the cells entered replicative senescence, which correlated with an increased expression of pericentromere transcripts. The pericentromeric heterochromatin was decondensed and exhibited reduced DNA methylation. Here, the produced transcripts may not have a specific biological role but rather be the consequence of a senescent state of the heterochromatin itself (Enukashvily et al., 2007). Aberrant overexpression of SatIII from 9q12 was observed in the Hutchinson–Gilford progeria syndrome (Shumaker et al., 2006). The latter arises from mutations in the laminA gene, which encodes a component of the nuclear lamina that maintains the structural integrity of the nucleus. Lamins are crucial for pericentromeric heterochromatin organization in interphase cells (Solovei et al., 2013).

Pericentromeric heterochromatin was show to lose H3K9me3 and HP1 proteins in older flies and human cells, leading to an anomalous expression of satellite sequences (Scaffidi and Misteli, 2006; Shumaker et al., 2006; Larson et al., 2012). Loss of pericentric silencing may drive age-related genome instability and death since the cells from older individuals or progeria patients are characterized by a global loss of heterochromatin marks, and increase in DNA damage (Scaffidi and Misteli, 2006; Shumaker et al., 2006; Leung et al., 2015). Inactivation of heterochromatin silencing components in flies cuts their lifespan in half whereas a moderate overexpression of HP1α extends their lifespan with 15%, suggesting that HP1α loss in older animals promotes aging (Larson et al., 2012). Finally, the transcriptional de-repression of satellite sequences has been linked to tau-induced neurodegeneration, as in Alzheimer’s disease (Frost et al., 2014).

### (Peri)centromere Transcription in Cancer and Disease

The transcriptional misregulation of the SatII and SatIII pericentromeric satellite sequences, and altered epigenetic state of pericentromeric chromatin characterizes many cancers and genetic disorders (Shumaker et al., 2006; Ehrlich, 2009; Eymery et al., 2009; Ting et al., 2011; Zhu et al., 2011; Figure 5B). In mouse models of pancreatic, colon, and lung cancers, satellite transcripts represent up to 50% of the total RNA, which was linked to deregulated DNA methylation. Specifically, in pancreatic ductal adenocarcinoma (PDAC) samples, 47% of all transcripts were produced from MajSat sequences. In contrast, in healthy reference tissues, only 0.02–0.4% of all transcripts originated from those repeats (Ting et al., 2011; Kishikawa et al., 2016a). The transcripts were highly heterogeneous (200–8,000 nt) and transcribed only from the forward strand. While PDAC murine cells expressed MajSats only minimally when cultured *ex vivo*, high expression levels similar to those observed in tumors *in vivo* were measured in immortalized PDAC tumor cells treated with 5-aza-2′-deoxycytidine, suggesting that transcription is regulated by DNA methylation, which might be re-established together with other epigenetic silencing mechanisms *ex vivo*. Furthermore, SatII expression showed a median 21-fold increase in human PDAC samples in comparison with “healthy” tissue samples (Ting et al., 2011). To determine what could be promoting SatII hyperexpression, linear regression analysis was performed to identify transcripts that were co-regulated with the mouse MajSat or human alpha-satellite sequences. Several genes involved in neuronal cell fate and stem cell pathways, that contained LINE1 transposable elements were highly expressed (Ting et al., 2011). A LINE1 insertion upstream of their transcription start sites can underlie their misregulation, contributing to cellular transformation. SatII RNA transcripts in colorectal cancer cells were reverse transcribed into DNA:RNA hybrids, and then generated dsDNAs, which were incorporated into the pericentromeric domains. Whole-genome sequencing showed that SatII copy number gain commonly characterizes human colon tumors, and is linked with low survival (Bersani et al., 2015). Healthy human testis tissue showed a high expression of pericentromeres, while in cancers their expression was silent (Eymery et al., 2009).

Methyltransferase DNMT3B, which methylates (peri)centromeric DNA at cytosines in CpG dinucleotides, is recruited by CENP-C. Impairment of this interaction causes an overproduction of CEN and pericentromere transcripts (Gopalakrishnan et al., 2009). Besides cancer, mutations in DNMT3B lead to the ICF syndrome (immunodeficiency, CEN instability, and facial anomalies) whose patients suffer from hypomethylated SatII and SatIII repeats (euchromatic gene methylation was at normal levels, Brun et al., 2011). The tumor-suppressing, heterochromatin-associating lysine demethylase 2A (KDM2A) is downregulated in prostate cancer (Frescas et al., 2008). Via its Jumonji domain, the enzyme demethylates the pericentromeric H3K36me2 modification to silence the heterochromatin. KDM2A depletion resulted in a loss of HP1 and elevated alpha-satellite and MajSat transcription in human and mouse cells, respectively. Phenotypes included genomic instability, sister chromatid misalignment, chromosome breaks, and anaphase bridges. The lower the level of KDM2A expression, the more severe the tumor grade in prostate cancer, linking hypermethylation and increased (peri)centromere transcription with cancer growth (Frescas et al., 2008; Figure 5B).

The histone demethylase JMJD2B acts as an oncogene in certain breast cancers (Slee et al., 2012). When overexpressed, its activity reduces H3K9me3 marks at CEN and causes chromosomal instability. While the levels of CEN and pericentromere transcripts in these tumors were not quantitated, their production was likely derepressed. A loss of SUV39H histone methyltransferase activity (mediates H3K9 methylation) facilitated the expression and/or stabilization of MinSat transcripts in mice, which accumulated as dsRNAs (Lehnertz et al., 2003; Martens et al., 2005). A forced accumulation of MinSat transcripts, in sense orientation, provoked a mislocalization of kinetochore proteins, affected chromosome segregation, sister chromatid cohesion, and induced modifications of CEN epigenetic hallmarks. Possibly, anomalous levels of CEN transcripts interfere with kinetochore and cohesin recruitment (Bouzinba-Segard et al., 2006). Of note, ectopic overexpression of alpha-satellite DNA in human cells led to chromosome loss but not to reduced methylation of the DNA. In contrast, DNA demethylation caused pathological alpha-satellite transcription and chromosome loss in human cells (Ichida et al., 2018).

SatII and SatIII transcripts were markedly overexpressed in human osteosarcoma cells depleted in tumor suppressor SIRT6, which deacetylates histone H3K16ac in pericentric heterochromatin. Its inactivation led to H3K18 hyperacetylation likely by the histone acetyltransferase GCN5, reversal of heterochromatin silencing, mitotic defects, genomic instability, and senescence. Importantly, depletion of the transcripts through RNAi rescued the phenotypes (Tasselli et al., 2016).

Mutations in the hereditary ovarian and breast cancer susceptibility gene BRCA1, which acts as a tumor suppressor, led to genomic instability. While BRCA1 acts in DNA replication and damage repair, control of the cell cycle, and many other regulatory functions, the protein was recently shown to also determine the epigenetic states of centromeric and pericentric chromatin (Zhu et al., 2011). Through its ubiquitin ligase activity, BRCA1 mono-ubiquitinates histone H2A at Lys119 (Chen et al., 2002; Figure 5B) to produce a repressive mark that prevents genomic instability and tumorigenesis (Zhu et al., 2011). When BRCA1 was knocked out in murine and human cells, a derepressed transcription of MinSat, MajSat, and alpha-satellite DNA was observed, respectively, concurrent with a loss of H2AK119 ubiquitination. While the latter may have produced defective heterochromatin (as indicated by reduced HP1 levels), it is unclear which factors promoted alpha-satellite transcription in the BRCA1-deficient cells. Ectopically expressing H2A fused to ubiquitin reversed the above BRCA1-loss phenotypes, whereas the ectopic expression of satellite DNA phenocopied it, resulting in DNA damage and genomic instability, cell cycle checkpoint defects, and centrosome amplification, indicating that overexpressed (peri)centromere transcripts could contribute to malignancy (Zhu et al., 2011).

The tumor-suppressing transcription factor Prep1 is associated with DNA damage control and the management of histone methylation levels (Iotti et al., 2011). Indeed, upon downregulating Prep1 in mouse or human cells, DNA damage increased. This phenotype, which is generated through an unknown mechanism, caused a widespread increase in the repressive histone mark H3K9me3. Consequently, the transcription of MajSat in mouse, and alpha-satellite DNA in humans dropped with 62% and 45%, respectively, compared to wild-type control cells. Intriguingly, the decrease in CEN and pericentromere transcript production led to the same phenotypes as in cells overexpressing them, including aneuploidy, miniature chromosomes, Robertsonian translocations, and CEN duplications (Iotti et al., 2011).

Tumor-suppressing transcription factor p53 cooperates with DNA methylation activity to silence a large part of the mouse genome. A massive transcription of major classes of retroelements, near-CEN tandem repeat satellite DNAs, and numerous species of non-coding RNAs was observed in p53-deficient mouse fibroblasts treated with 5-aza-2′-deoxycytidine (not observed in treated p53 wild-type cells). The levels of these transcripts exceeded those of β-actin mRNA by more than 150-fold. Accumulation of these transcripts, which are capable of forming dsRNAs, was complemented by a potent apoptosis-inducing type I interferon response. The authors suggested a model in which the downregulation of these repeat sequences is controlled by p53-driven transcriptional silencing, DNA methylation-based suppression of transcription, and the suicidal type I interferon response, which eliminates the cells that escaped the first two lines of control (Leonova et al., 2013).

(Peri)centromere silencing is also regulated by the Polycomb repressive complexes PRC1 and PRC2, which are commonly misregulated in cancer (Blackledge et al., 2015). PRC2 lysine methyltransferase subunit EZH2 catalyzes the addition of one to three methyl groups to histone H3 at Lys27 (Figure 5B). In *Rb1* mutant mice, which are defective in recruiting EZH2 to repetitive sequences, a transcriptional derepression of satellite DNA was observed, which induced susceptibility to lymphoma (Ishak et al., 2016).

## (Peri)Centromere Transcripts as Cancer Biomarkers and Targets in the Clinic

SatII overexpression characterizes myriad cancerous and precancerous lesions, suggesting that SatII RNA levels might be a good predictor or indicator of cancer (Ting et al., 2011; Bersani et al., 2015; Tasselli et al., 2016; Hall et al., 2017). Indeed, RNA *in situ* hybridization analysis of SatII expression in biopsies proved a better diagnostic for pancreatic cancer than standard histopathological analysis (Ting et al., 2011). A convenient and highly sensitive method for quantitating circulating satellite repeat RNAs in blood serum (Kishikawa et al., 2016b) combines Tandem Repeat Amplification by nuclease Protector (TRAP) with droplet digital PCR. Patients with pancreatic ductal carcinoma (PDAC) were efficiently discriminated from healthy individuals, while patients with intraductal papillary mucinous neoplasm, a precancerous lesion of PDAC, could also be accurately identified. This simple and cheap test allows for early prognosis, quick screens, and regular follow-ups of PDAC progression. This method may well be adapted to quantitate additional (peri)centromere transcripts in other cancers as well.

Kinetochore subunit overexpression (Thiru et al., 2014; Zhang et al., 2016; Sun et al., 2016), CENP-A overproduction and mislocalization (Athwal et al., 2015), and de-silenced (peri)centromeric chromatin may all contribute to aneuploidy and promote cancer initiation/progression. The degree of overexpression of kinetochore protein-encoding genes, which associate with patient survival and response to therapy, could classify tumors, and serve as future prognostic cancer biomarkers (O’Brien et al., 2007; Sun et al., 2016; Zhang et al., 2016). Similarly, CEN and pericentromere transcript levels in conjunction with the (peri)centromeric methylation, acetylation, or ubiquitination state may serve as valuable readouts of cancer grade and survival. They may also represent novel therapeutic targets. In fact, various drugs inhibiting numerous epigenetic enzymes/regulators are in advanced developmental stages (Pfister and Ashworth, 2017). Nucleic acid therapeutics aimed at (peri)centromere repeats may provide alternative objectives for the future. They are transcribed in a cell- or tissue-specific manner, making them exceptional objectives. Powerful RNA structure determination assays can also map the secondary and tertiary structure of these RNAs (Wilkinson et al., 2006; Novikova et al., 2013; Lu et al., 2016). Clinical trials are already underway to similarly target highly structured bacterial or viral riboswitches using small-molecule inhibitors to treat bacterial and viral infections, respectively (Howe et al., 2015). Small-molecule ligands targeting structural elements in these CEN or pericentromere RNAs could potentially destabilize the transcript or interfere allosterically with CEN-protein binding to confer a therapeutic effect, although this remains purely hypothetical. With recent advances in genome editing methods, it is possible to achieve transcriptional silencing of (peri)centromere repeats via CRISPR interference (Gilbert et al., 2014; Koch, 2017). In a genome-wide CRISPR interference study, guide RNAs were developed to selectively and successfully inactivate >16,000 long non-coding RNA genes within the human genome (Liu et al., 2017). These experiments suggest that downregulating pathologically expressed (peri)centromeric elements could well be feasible (Zhang et al., 2016; Koch, 2017). The recently developed CRISPR/Cas13 system (Abudayyeh et al., 2017) represents another promising approach to knock down non-coding RNAs. However, only the future will tell to which extent these approaches will translate into clinical scenarios.

## Perspectives

The continuous identification and functional characterization of new epigenetic activities (enzymes, histone modifications) that impinge on centromeric and pericentromere domains via ever more sensitive mass spectrometry approaches will take our understanding of CEN, kinetochore, and pericentromere biology to the next level. In addition, transcription factors that drive (peri)centromere transcription in healthy and diseased cells must be identified as their biology and influences on the spatiotemporal regulation of the CEN and pericentric regions remains largely unknown. The same is true for regulators that act upon the RNAPII complex to orchestrate its activity (recruitment, elongation, termination) at (peri)centromeres. Kinases and phosphatase may be prime candidates. A better understanding of RNAi pathway involvement in mammalian biology would be welcomed as well. For sure, exciting (peri)centromere biology will continue to be “written” in laboratories worldwide, and hopefully at some point in cancer clinics as well.

## Author Contributions

KS and PDW wrote the paper and made the figures. Both authors have approved of the manuscript.

## Conflict of Interest Statement

The authors declare that the research was conducted in the absence of any commercial or financial relationships that could be construed as a potential conflict of interest.
